# The PANDA Unit: Responding to complexity and comorbidity in acute mental health

**DOI:** 10.1192/j.eurpsy.2023.1096

**Published:** 2023-07-19

**Authors:** J. Brett, J. Huber, G. Aguirrebarrena, P. T. Preisz

**Affiliations:** 1 Clinical Pharmacology, St. Vincents Hospital; 2 Mental Health; 3 Emergency Medicine, St. Vincent’s Hospital; 4 Emergency Medicine, St. Vincents Hospital, Sydney, Australia

## Abstract

**Introduction:**

Acute mental health presentations such as suicidality or psychosis are frequently accompanied by intoxication, substance use disorder, deliberate self-poisoning and social crises. There is a need to break down silos and provide integrated multi-disciplinary acute care for such individuals.

**Objectives:**

Here we describe outcomes of the Psychiatry And Drug ANd Alcohol (PANDA) Unit, a unique physical space collocated with the emergency department (ED) along with a unique model of care, providing multidisciplinary care to individuals presenting with acute mental health concerns plus complex comorbidity.

**Methods:**

A description of the PANDA model of care and Service characteristics along with process and outcome measures across the first 12 months that the PANDA Unit was operational. These include number of patients admitted, patient demographics and characteristics, length of stay, referral destinations and impact on the occurrence of behavioural disturbance across the ED.

**Results:**

PANDA opened in November 2020 and since then has admitted an average of 122 patients per month (15% Aboriginal) with an average bed occupancy of 79% and average length of stay of 1.3 days. An average of 12% of patients were scheduled under the mental health act and an average of two patients were stepped down from ICU each month, with the remainder being admitted via the ED. An average of 80.7% of these were discharged home directly while 7.2% were transferred for inpatient withdrawal management and 8.9% to inpatient mental health services. The top three substances of concern were alcohol methamphetamine and heroin and an average of 16.5% of people reported injecting drugs in the prior 3 months. An average of 37.8% patients had been seen by an emergency physician and admitted to the PANDA Unit within 4 hours, one of the best performing units hospital-wide. In the 6 months prior to PANDA becoming operational there was a median of 20 episodes of behavioural disturbance requiring restraint per month across the ED. This dropped to 12 episodes in the six months following the PANDA Unit opening.

**Conclusions:**

The PANDA Unit model of care has proven feasible to implement and has made a positive impact on a previously underserved patient population. It has also contributed to improving the integration of care for mental health patients with comorbidity as well as reducing behavioural disturbance and improving patient flow across the emergency department.

**Disclosure of Interest:**

None Declared

